# Excision of the Eyeball

**Published:** 1899-04

**Authors:** 


Excision of the Eyeball. Excision of the eyeball becomes a frequent necessity in a dog-clinic in cases of laceration or extreme dislocation. In the latter case excision should not be too quickly resorted to. Even when pulled out of its socket on the side of the cheek the eyeball, when not itself injured, may be replaced into its socket with careful manipulation and maintained by pressure. Sometimes it may be necessary to slit the external canthus of the eyelids in order to replace the eye. When removal of the eyeball is necessary, it should not be removed in its entirety by severing, with the scissors, the muscles that surround the eye globe, which is, perhaps, the most frequent custom.

A better method consists in making a circular incision through the sclerotic about an eighth of an inch from the periphery of the cornea. The ocular contents will readily escape, and the retina and choroid can be scraped from the inside of the sclerotic. The latter will collapse, and, though not very vascular, its inside will granulate and the partially obliterated cavity of the sclerotic will soon fill up and cicatrize. Beside there is less pain and suppuration, and less time required for cicatrization after this procedure than when the nerves and muscles themselves are severed.

